# Analysis of outcomes following loop electrosurgical excision and clinical features of patients with cervical high-grade squamous intraepithelial lesions with abnormal preoperative endocervical curettage

**DOI:** 10.1186/s12957-023-03088-5

**Published:** 2023-08-03

**Authors:** Chunyang Feng, Liying Gu, Yingting Wei, Jiaxin Niu, Haima Yang, Zubei Hong, Lihua Qiu

**Affiliations:** 1 Department of Obstetrics and Gynecology, Ren Ji Hospital, Shanghai Jiao Tong University School of Medicine, Shanghai, 200127 China; 2 Shanghai Key Laboratory of Gynecologic Oncology, Shanghai, 200127 China; 3 School of Optical-Electrical and Computer Engineering, University of Shanghai for Science and Technology, Shanghai, 200093 China; 4 State Key Laboratory of Oncogenes and Related Genes, Shanghai Cancer Institute, Shanghai, 200127 China

**Keywords:** Endocervical curettage, High-grade squamous intraepithelial lesions, Human papillomavirus, Loop electrosurgical excision procedure, Risk factors

## Abstract

**Objective:**

The purpose of this study was to identify the clinical characteristics of patients with high-grade squamous intraepithelial lesions (HSIL) with abnormal endocervical curettage (ECC) and to evaluate the efficacy of abnormal preoperative ECC in predicting recurrence after a loop electrosurgical excision procedure (LEEP).

**Methods:**

We retrospectively analyzed a total of 210 cases of histological HSIL in female patients diagnosed using cervical biopsy and/or indiscriminating ECC, and these included 137 cases with normal ECC and 63 cases with abnormal ECC. We also collected preoperative information and data on postoperative human papillomavirus (HPV) and histological outcomes within 2 years.

**Results:**

The additional detection rate of HSIL using indiscriminating ECC was 5%. Patients with abnormal ECC were older (*P* < 0.001), predominantly menopausal (*P* = 0.001), had high-grade cytology (*P* = 0.032), a type 3 transformation zone (*P* = 0.046), and a higher proportion of HPV type 16/18 infection (*P* = 0.023). Moreover, age (odds ratio [OR] = 1.078, 95% confidence interval [CI] = 1.0325–1.1333, *P* = 0.003) and HPV 16/18 infection (OR = 2.082, 95% CI = 1.042–4.2163, *P* = 0.038) were independent risk factors for abnormal ECC. With an observed residual lesion/recurrence rate of 9.5% over the 24-month follow-up, we noted a 9.3% higher rate in the abnormal ECC group when compared with the normal ECC group. Abnormal preoperative ECC (OR = 4.06, 95% CI = 1.09–15.14, *P* = 0.037) and positive HPV at the 12-month follow-up (OR = 16.55, 95% CI = 3.54–77.37, *P* = 0.000) were independent risk factors for residual disease/recurrence.

**Conclusion:**

Preoperative ECC was one of the risk factors for post-LEEP residual/recurrent HSIL, and detecting abnormal ECC when managing older patients or patients with HPV 16/18 infection during colposcopy is critical.

## Introduction

Cervical cancer ranks fourth among all malignant tumors in women in terms of the incidence and fatality rate, according to global cancer data, implying that effective prevention and treatment are critical [1]. Persistent human papillomavirus (HPV) infection is a risk factor for cervical cancer and its precancerous changes. Vaccination against HPV is an essential measure of primary prevention for cervical cancer, and its introduction has stabilized the incidence of cervical cancer in developed countries. However, even in the USA, where HPV vaccination rates are high, the probability of developing cervical cancer was 0.3% in women under 49 years of age and 0.1–0.2% in women over 50 years in 2016–2019 [2]. Proven secondary prevention measures include early diagnosis and treatment of precancerous lesions [3]. High-grade squamous intraepithelial lesions (HSIL) are particularly important given their high risk of progression, with the attendant incidence rate documented to be 1–2% in a large population study [4]. The diagnosis of HSIL is mainly based on the histological evaluation of cervical biopsy specimens obtained under colposcopy.

Endocervical curettage (ECC) plays a significant role in evaluating lesions in the entire cervix and has an extensive range of clinical applications. The 2012 and 2019 American Society for Colposcopy and Cervical Pathology (ASCCP) guidelines recommend endocervical curettage (ECC) for incompletely visible transformation areas, stating that ECC is also acceptable for visible lesions [5, 6]. When compared with ectocervical biopsy alone, the combination of ECC and ectocervical biopsy can improve the detection rate of HSIL by an additional 1.1–11.9% [7–9]. As loop electrosurgical excision procedure (LEEP) is one of the most commonly used methods for the treatment of HSIL, relationship between ECC and different perioperative periods of LEEP attracted attention in clinic. It was previously reported that 31% of cases of recurrent/residual disease were diagnosed using ECC during the follow-up period following a LEEP [10]. There was another study which recognized ECC during LEEP surgery as an indicator of insider status, found that intraoperative ECC was one of the risk factors of post-LEEP recurrent/residual [11]. However, few studies reported the relationship between preoperative ECC and recurrent/residual HSIL after LEEP.

Although ECC plays an important role in the preoperative diagnosis and postoperative follow-up of HSIL, it is still associated with various potential complications. Firstly, as a relatively invasive procedure in which the curette is inserted deep into the cervical canal, ECC is associated with pain and discomfort, which may lead to poor cooperation during the procedure. Secondly, inadequate sampling in the ECC procedure may cause the correct pathology to be missed. Furthermore, the population of women most likely to benefit from ECC has yet to be identified. Lastly, the relationship between preoperative ECC and post-LEEP recurrent/residual HSIL remains unclear.

In this context, we designed this research to address the following two purposes: first, to identify characteristics of HSIL with abnormal preoperative ECC; and second, to evaluate the utility of abnormal preoperative ECC in predicting HSIL recurrence following LEEP.

## Materials and methods

This was a retrospective study involving 358 women with histologically confirmed HSIL (ICD code 2E66.2) who underwent colposcopy-guided biopsy and ECC at the Renji Hospital between June 2018 and June 2019. We excluded women who did not consent to LEEP treatment, had post-LEEP invasive pathology, had positive margins on postoperative pathology, had missing data regarding preoperative general clinical information or postoperative follow-up data at 4–6 months, 12 months, and 24 months, and those who underwent hysterectomy within 24 months following LEEP. Finally, a total of 200 patients were included for analysis. This study was approved by the Ethics Committee of Ren Ji Hospital (reference KY2020-018). The consent was obtained the patient was informed the details about study design. For personal information protection, each patient was assigned a unique code for matching medical data. The data was de-identified and shared only among those involved in the trial.

### Patient information

We evaluated the following clinical features: age, menstrual status (post-menopause/menstruation), anamnesis, type of transformation zone (TZ), cytology, and HPV status preoperatively and at 4–6, 12, and 24 months postoperatively. Anamnesis was divided into infection history, lesion history, and none/unknown, with infection history defined as persistent infection with high-risk HPV (HR-HPV) for more than 6 months prior to the histopathological diagnosis of HSIL, and no history of cervical lesions in the past; and lesion history was defined as a history of cervical lesions (including treatment history) with a histopathological diagnosis of squamous intraepithelial lesions (SILs) for more than 6 months prior to the preoperative colposcopy.

### Cytology diagnosis

Cytology results were reported per the Bethesda system: negative for intraepithelial lesion or malignancy (NILM), atypical squamous cells of undetermined significance (ASCUS), Low-grade squamous intraepithelial lesions (LSIL), atypical glandular cells (AGC), HSIL, squamous cells cannot exclude high-grade squamous intraepithelial lesions (ASC-H). Further, they were classified as low-grade (NILM, ASCUS, LSIL,AGC), and high-grade (HSIL, ASC-H) [5].

### Human papillomavirus test

In accordance with WHO recommendations for cervical screening, HPV-DNA was used for HPV testing. The test was performed in department of docimology, and the 21 HPV genotyping kit diagnostic kit was from Hybribio Ltd., Hong Kong, which was based on PCR-low density gene microarray and inflow hybridization technology. According to “Global HPV DNA PP 2021 technical report” published by WHO, the accuracy of this kit is 100%. In this study, we focused only on high-risk HPV (HR-HPV). According to the results, classification of 21 types of HPV was listed here: high-risk HPV was defined as HPV types 16, 18, 31, 33, 35, 39, 45, 51, 52, 53, 56, 58, 59, 66, and 68 (a total of 15 genotypes), while low-risk HPV was defined as HPV types 6, 11, 42, 43, 44, and 81 (a total of six genotypes). We classified the HPV status into three categories: HPV 16/18, other HR-HPV, and negative. One patient who had both HPV 16/18 and other HR-HPV types was included in the HPV 16/18 group.

### Pathology

All tissue samples were sent to the pathology department for histological analysis, and all specimens were independently reviewed by two experienced pathologists with senior title. The lesions were diagnosed as HSIL or LSIL according to the 2014 classification criteria of the World Health Organization (WHO). LSIL: hyperplasia of squamous epithelial basal and parabasal-like cell, mildly disturbed nuclear polarity, mild anisotropy, few karyomitosis, lesion location was limited to 1/3 of subepithelial, and negative p16 staining or scattered dotted positivity in the epithelium. HSIL: chaos nuclear polarity, increased ratio of nuclear and plasma, increased nuclear karyomitosis, atypia cells extending over 1/3 of subepithelial layer or even the whole layer, and diffusing and continuous p16 staining distributing over field of 2/3 epithelial layer. Ectocervix and canal tissue samples were also collected during the colposcopy procedure. The outcomes following LEEP were classified into the following categories: (1) cure (no evidence of cervical lesions on cervical biopsy and ECC under colposcopy within the 24-month follow-up); (2) residual disease (any evidence of SILs on cervical biopsy and ECC within 4–6 months following LEEP); and (3) recurrence (no evidence of SILs via cervical biopsy and ECC within 4–6 months following LEEP, but evidence of SILs at more than 6 months postoperatively) [10, 12, 13].

### Follow-up strategy and outcome

Patients underwent a full evaluation consisting of an HPV test, a ThinPrep cytology test, colposcopy-guided biopsy, and ECC at 4–6 months postoperatively, and HPV and cytology screening were subsequently performed at 12 and 24 months following LEEP. Any patients with abnormal screening results underwent colposcopy-guided biopsy and ECC for further diagnosis at the histology level. The overall outcome of this study was residual/recurrent HSIL within 24 months following LEEP.

### Statistical analyses

The data were analyzed using IBM SPSS statistics software (version 19). Non-normal distribution measurement data were represented using P50 (P25–P75), and the non-parametric rank-sum test was used for inter-group comparisons. Enumeration data were expressed as *N* (%), and inter-group comparisons were done using the chi-square (*χ*^*2*^*)* test. A multivariate logistic regression model was used to analyze the risk factors for abnormal preoperative ECC and residual disease/recurrence within 24 months. A *P*-value of < 0.05 was considered statistically significant.

## Results

### General characteristics

The final analysis consisted of a total of 200 women patients. The mean age was 38 years (32.3–46), and a minority of them (17%) were post-menopausal. In the cytology evaluation, 37 patients (20%) were diagnosed as high-grade, 92 patients (46%) had HPV16/18 infection, and 97 patients (48.5%) had other HR-HPV infections. As for TZ, 89 patients (44.5%) had a type 3 TZ, and 78 (39%) patients had a type 1 TZ. 63 patients (31.5%) had abnormal preoperative ECC, and 137 (68.5%) had normal preoperative ECC (Table 1). Unless the patient is pregnant, ECC is performed as a routine procedure during colposcopy in our clinic.

**Table 1:** General background of included population

Characteristics	Classification	P50(P25-P75)/*N*(%)
Age/years old		38(32.3~46)
Menstrual status	Menstruation	166(83.0)
	Post-menopause	34(17.0)
Anamnesis	None/unknown	156(78.0)
	Infection history	26(13.0)
	Lesion history	18(9.0)
Cytology	≤Low grade^a^	148(80.0)
	High grade^b^	37(20.0)
HPV	Negative	11(5.5)
	HPV16/18	92(46.0)
	Other HR-HPV	97(48.5)
TZ	1	78(39.0)
	2	33(16.5)
	3	89(44.5)
Biopsy	Normal	7(3.5)
	LSIL	4(2.0)
	HSIL	189(84.5)
ECC	Normal	137(68.5)
	Abnormal	63(31.5)

*Abbreviations*: *HPV* Human papillomavirus, *HR-HPV* High-risk HPV, *TZ* Transformation zone, *LSIL* Low-grade squamous intraepithelial lesion, *HSIL* High-grade squamous intraepithelial lesion, *ECC* Endocervical curettage

^a^Low-grade: negative for intraepithelial lesion or malignancy, atypical squamous cells of undetermined significance, low-grade SIL, atypical glandular cells

^b^High-grade: HSIL, squamous cells-cannot exclude high-grade squamous intraepithelial lesion

The pathology analysis of the biopsy and ECC is shown in Table 2. In the sample, it was possible to diagnose HSIL in 58 patients (29%) using ECC alone, while cervical biopsy was used for diagnosis in 189 (94.5%). As 6 patients with normal ectocervical biopsy and 4 patients with biopsy had HSIL of ECC, the HSIL diagnosis rate using the combined method was 99.5% ((189 + 6 + 4)/200); with this, the additional detection rate of HSIL via ECC was 5% ((6 + 4)/200) (Table 2). It was notable that one patient diagnosed with a normal cervical biopsy and LSIL using ECC was diagnosed with HSIL following LEEP. This patient was a 63-year-old post-menopausal woman with a history of LEEP for HSIL, negative HPV status, and abnormal cytology (atypical squamous cells of undetermined significance). While the patient had a type 3 TZ, the colposcopy procedure indicated invasive cancer, and diagnostic conization was thus recommended despite the diagnosis of LSIL via ECC, and the final diagnosis was HSIL.

**Table 2:** Pathology of biopsy and ECC

		Biopsy			*N*(%)
		Normal	LSIL	HSIL	
	Normal	0	0	137	137(68.5)
ECC	LSIL	1	0	4	5(2.5)
	HSIL	6	4	48	58(29.0)
*N*(%)		7(3.5)	4(2.0)	189(94.5)	200

### Characteristics of patients with abnormal endocervical curettage

We compared the following clinical features between the normal ECC and abnormal ECC groups: age, menstruation, anamnesis, HPV, cytology, and TZ. As shown in Table 3, the women in the abnormal ECC group were 8 years older than women in the normal ECC group (44 [37.0–51.0] vs. 36.0 [31.0–42.0] years, respectively; *P* < 0.001). The post-menopausal rate of patients in the abnormal ECC group was 19.3% higher than that of normal ECC group patients (30.2% vs. 10.9%, respectively; χ2 = 11.286, *P* = 0.001). Compared with patients in the normal ECC group, those in the abnormal ECC group had a 7% higher HPV 16/18 infection rate and a 12.7% lower infection rate of other HR-HPV types (50.8% vs. 43.8%, 38.1% vs. 50.8%, respectively; *P* = 0.023). The proportion of patients with high-grade cytology in the abnormal ECC group was 13.6% higher than those in the normal group (29.3% vs. 15.7%, respectively; *P* = 0.032). The proportion of patients with type 3 TZ in the abnormal ECC group was 18.4% higher than that of patients in the normal group (57.1% vs. 38.7%, respectively; *P* = 0.046). There was no significant difference in the frequency of anamnesis between the two groups (*P* = 0.208).

**Table 3:** Clinical characteristics of abnormal ECC

Characteristics		Normal ECC	Abnormal ECC	*χ*^*2*^	*P*	OR
		*n* = 137	*n* = 63			
Age/years old		36.0(31.0–42.0)	44(37.0–51.0)	/	0.000	/
Menstrual status	Post-menopause	15(10.9)	19(30.2)	11.286	0.001	3.512
	Menstruation	122(89.1)	44(69.8)			
Anamnesis	None/Unknown	102(74.5)	54(85.7)	3.156	0.208	/
	Infection History	20(14.6)	6(9.5)			
	Lesion History	15(10.9)	3(4.8)			
HPV	HPV16/18	60(43.8)	32(50.8)	7.409	0.023	1.307
	Other HR-HPV	72(50.8)	24(38.1)			
	Negative	4(2.9)	7(11.1)			
Cytology	High grade	20(15.7)	17(29.3)	4.577	0.032	2.218
	≤Low grade	107(84.3)	41(70.7)			
TZ	3	53(38.7)	36(57.1)	6.142	0.046	1.848
	2	24(17.5)	9(14.3)			
	1	60(43.8)	18(28.6)			

### Multivariate analysis of risk factors for abnormal preoperative endocervical curettage

In the multivariate logistic regression analysis, we took abnormal ECC as the dependent variable and included the following clinical characteristics that showed statistically significant differences in the univariate analysis: age, menstruation, TZ, cytology, and HPV (Table 4). The results indicated that age was an independent risk factor for abnormal ECC (odds ratio [OR] = 1.078, 95% confidence interval [CI] = 1.025–1.133, *P* = 0.003). Compared with other HR-HPV alone, HPV 16/18 (OR = 2.082, 95% CI = 1.042–4.163, *P* = 0.038) and negative HPV (OR = 6.413, 95% CI = 1.512–27.363, *P* = 0.012) were independent risk factors. Other clinical variables, such as menstruation, TZ, and cytology, could not be considered independent risk factors.

**Table 4:** Multivariate analysis of abnormal preoperative ECC (Abnormal ECC = 1)

Factors		*β*	*SE*	*Wald*	*P*	OR (95%CI)
Age/years old		0.075	0.026	8.579	0.003	1.078(1.025,1.133)
Menstrual status	Post-menopause					1.000
	Menstruation	− 0.271	0.620	0.191	0.662	0.762(0.226,2.569)
TZ	1					1.000
	2	− 0.010	0.520	0.000	0.985	0.990(0.357,2.746)
	3	0.157	0.420	0.140	0.709	1.170(0.513,2.666)
Cytology	≤ Low grade					1.000
	High grade	0.679	0.418	2.640	0.104	1.972(0.869, 4.473)
HPV	Negative					1.000
	HPV16/18	0.734	0.353	4.309	0.038	2.082(1.042,4.163)
	Other HR-HPV	1.861	0.739	6.346	0.012	6.431(1.512,27.363)

### Disease outcomes following loop electrosurgical excision

**Fig. 1:**
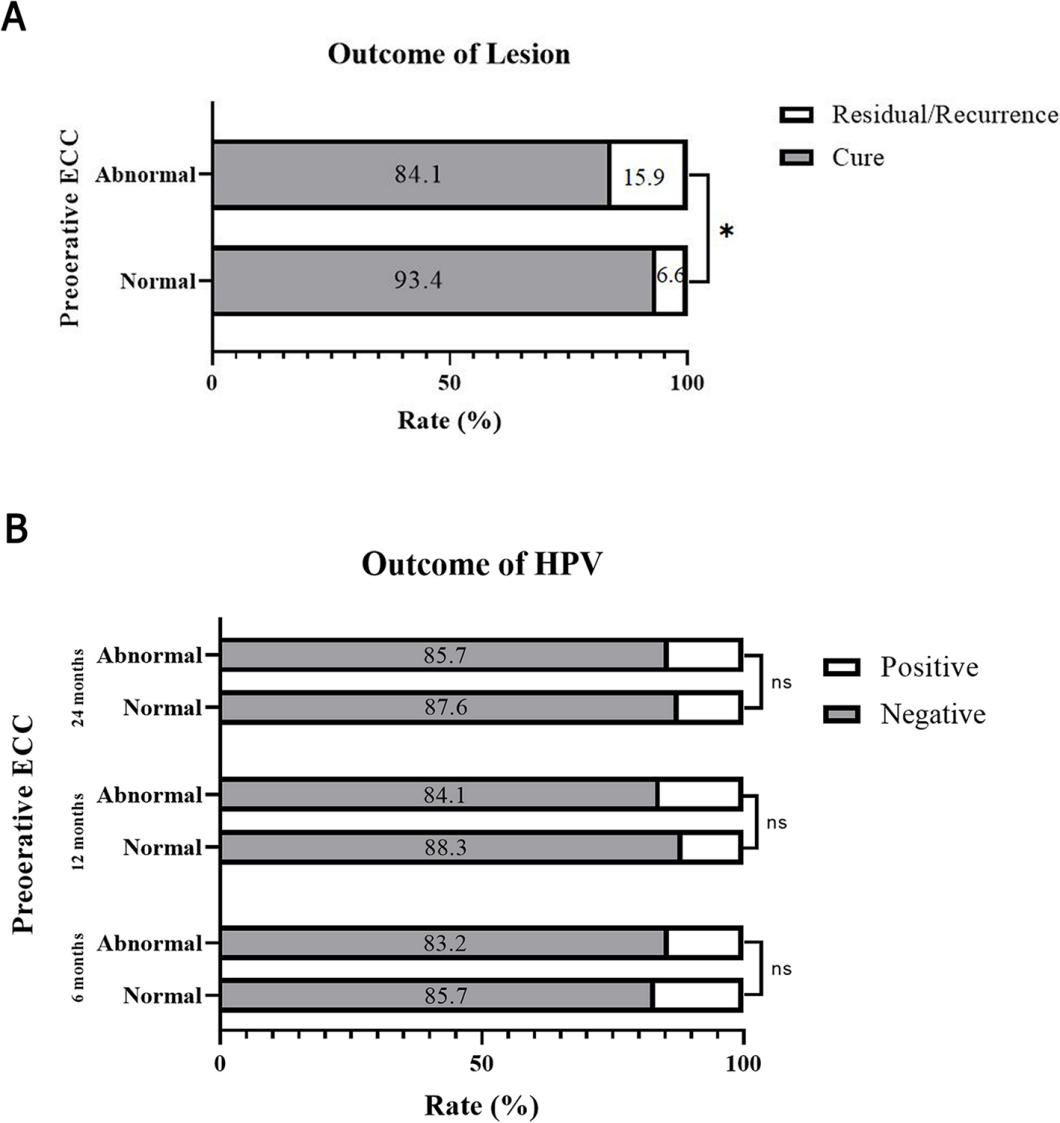
Lesion and HPV Outcomes within 24 months after LEEP. **A** The overall residual disease/recurrence rate was significantly higher in patients in the abnormal ECC group than those in the normal ECC group (15.9% vs. 6.6%, respectively; *P* = 0.037, RR = 2.409). **B** The HPV negative conversion rates at the follow-up points of 6, 12, and 24 months were 84.0%, 87.0%, and 87.0%, respectively, with no significant difference between the two groups (85.7% vs. 83.2%, *P* = 0.654; 84.1% vs. 88.3%, *P* = 0.413, and 85.7% vs. 87.6%, *P* = 0.714, respectively)

All the patients were followed up for 24 months, and the disease outcomes were evaluated (Fig. 1). The overall residual disease/recurrence rate was 9.5%, and this was significantly higher in patients in the abnormal ECC group than those in the normal ECC group (15.9% vs. 6.6%, respectively; *χ*^*2*^ = 4.345, *P* = 0.037, RR = 2.409), which was shown in Fig. 1A. As shown in Fig. 1B, the HPV negative conversion rates at the follow-up points of 6, 12, and 24 months were 84.0%, 87.0%, and 87.0%, respectively, with no significant difference between the two groups (85.7% vs. 83.2%, *P* = 0.654; 84.1% vs. 88.3%, *P* = 0.413, and 85.7% vs. 87.6%, *P* = 0.714, respectively).

We used univariate logistic regression analysis to compare the following clinical features between different outcomes within 24 months: age, menstrual status, preoperative HPV, cytology, ECC, and postoperative HPV at the follow-up points. We found that there were significant differences in menstrual status (*P* = 0.021), ECC (*P* = 0.043), and postoperative HPV at 6, 12, and 24 months (*P* = 0.000, 0.000, and 0.000, respectively). We further conducted a multivariate logistic regression analysis to filter the features with significant differences in the univariate analysis. The results indicated that abnormal preoperative ECC (OR = 4.06, 95% CI = 1.09–15.14, *P* = 0.037) and positive HPV at the 12-month follow-up (OR = 16.55, 95% CI = 3.54–77.37, *P* = 0.000) were independent risk factors for residual disease/recurrence (Table 5).

**Table 5:** Univariate and multivariate analysis of postoperative outcome

Characteristics	Cure	Residual/recurrence	*P*	*P’*	OR (95%CI)
	*n* = 181	*n* = 19			
Age	38.0(32.0–45.0)	42.0(37.0–52.0)	0.055		
Menstrual status					
Menstruation	154	12			
Post-menopause menopause	27	7	0.021	0.386	1.89(0.45–7.93)
HPV					
Negative	11	0			
HPV16/18	88	9	0.999		
Other HR-HPV	82	10	0.999		
Cytology					
≤ Low grade	35	2			
High grade	146	17	0.356		
ECC					
Normal	128	9			
Abnormal	53	10	0.043	0.037	4.06(1.09–15.14)
HPV-6 months					
Negative	162	6			
Positive	19	13	0.000	0.065	4.50(0.91–22.15)
HPV-12 months					
Negative	169	5			
Positive	12	14	0.000	0.000	16.55(3.54–77.37)
HPV-24 months					
Negative	166	8			
Positive	15	11	0.000	0.520	1.71(0.34–8.70)

*P* univariate analysis. *P’* multivariate analysis

## Discussion

The ECC method is an effective technique for evaluating cervical lesions, potentially increasing the detection rate of cervical lesions under colposcopy, making it significant for future clinical decision-making. Because of the variety of application scenarios, the HSIL detection rate of ECC and the additional detection rate are often variable. Current studies demonstrate that the HSIL detection rate using ECC is 1.0–34.9% and that the additional detection rate is 1.1–11.9% [7–9, 14]. Many factors influence the detection rates and additional HSIL detection rates when using ECC, and these include demographic characteristics, testing time, and analytical views.

In the current study, of all the female patients with HSIL, 29% were detected via routine ECC. We found that, when compared with cervical biopsy alone, ECC increased the additional detection rate of HSIL by 5%, and this is consistent with previous reports. This improved detection rate of cervical cancer using ECC has obvious clinical significance. Furthermore, when using ECC, there have been cases of extra-early diagnoses of cervical adenocarcinoma with pelvic lymph node metastasis [9, 15, 16]. As a result, it is evident that more attention should be placed on the use of ECC in clinical practice.

However, because it is an invasive procedure in which the curette is inserted deep into the cervical canal, ECC can result in discomfort for the patient during the examination, while the method also entails the risk of obtaining poor-quality samples. Therefore, on the one hand, for patients who have to undergo ECC, we try to reduce patients’ pain as much as possible through adequate humanistic care, gentle operation and local anesthesia when necessary. On the other hand, the quality of the samples was improved by using gauze to collect samples in the posterior fornix, and sufficiently rotating the curette in the fixative to prevent tissue missing. Furthermore, as part of this study, an attempt was made to identify patients who are most likely to benefit from ECC to narrow the population.

In this paper, we summarized the characteristics of patients with abnormal ECC in a routine ECC sample. First of all, for the type 2–3 transformation zone, the squamo-columnar junction that predisposes to cervical lesions is partially or completely located in the cervical canal, which is consistent with the location to be assessed by ECC. Considering that the estrogenic change influenced by age and menopausal status affects the type of transformation zone and the local immune of the cervix, we included them in the clinical factors to be explored. In addition, HPV and TCT are essential indicators of cervical screening, so these factors were taken into account. Considering the potential interplay between these factors, multivariate regression analysis was used to eliminate confounding factors. Univariate analysis results showed that patients with HSIL and abnormal ECC were comparatively older and post-menopausal and had HPV 16/18 infection, high-grade cytology, and a type 3 TZ. In clinical practice, women with the mentioned characteristics should be evaluated for the possibility of abnormal ECC. Additionally, multivariate regression analysis revealed that age and HPV 16/18 were independent influencing factors of abnormal ECC, with the risk of abnormal ECC increasing by 1.078× for every 1-year increase in age, while the risk increases 2.082× with HPV 16/18 infection when compared with other HR-HPV infections. As a result, the use of ECC is especially beneficial in older patients with HPV 16/18 infections.

However, in this study, we did not identify the age cutoff for abnormal ECC, and this aspect warrants further investigation. Furthermore, the age criteria also need to be clarified in the ASCCP guidelines. At present, the guidelines recommend ECC for women with atypical glandular cells and adenocarcinoma in situ cytology, and when the TZ is not completely visible under colposcopy [5, 6]. Liu et al. found that the diagnosis rate of HSIL using ECC increases with an increase in age among women aged 30 years or older [14]. Greater attention should be paid to ECC for women over 45 years with persistent HPV 16 infection, while indications for ECC in women under 30 years of age include abnormal cytological gland cells, partially visible TZ, and high-grade cytology that is inconsistent with normal coloscopy. So, to confirm the age cutoff of abnormal ECC, large-scale studies involving patients of multiple ages are required. Perhaps we can group the patients participating in the study into different age groups and calculate the probability of abnormal ECC in each group separately. Additionally, we believe that there may be more than one age cutoff for abnormal ECC due to geographical, ethnic, and occupational factors.

Liu et al. [14] also found that the diagnosis rate of HSIL using ECC was higher in women with HPV 16/18 and high-grade cytology. Another study [17] related to conventional ECC found that factors such as a completely visible TZ, age under 45 years, a history of contraceptive exposure, premenopausal status, and having fewer than four children reduced the additional diagnostic utility of ECC. The additional diagnosis of HSIL using ECC was more likely in women who were dissatisfied with the results of colposcopy, were older, had no history of contraceptive exposure, were menopausal, and had more than four children. However, the latter study did not confirm the final pathology via LEEP [17]. Based on the above studies, our results pertaining to the features of patients in our study that could benefit from ECC are comparable to those of previous research.

Conization is the main treatment method of HSIL, which can be achieved by cold knife or LEEP. Even though LEEP has some limitations, such as the thermal burn effect which may affect the pathological diagnosis of the incisal margin tissue, and the control of the excising depth by the electric knife is slightly weaker due to shape limitation of electric knife. LEEP is still extensively used for HSIL treatment because of its simplicity of use, lower cost reduction, and fewer postoperative complications [18]. The incidence of persistence/recurrence of HSIL following LEEP ranges from 2.8 to 11.3%, and this is influenced by several factors, including demographic characteristics, follow-up strategy, and the definition of residual/recurrent disease [19–21]. In the current study, we found that the overall residual disease/recurrence rate at 24 months following surgery was 9.5%, which was consistent with previous reports. The risk factors for persistence/recurrence include age over 45 years, positive resection margins, abnormal intraoperative ECC, persistent postoperative HR-HPV infection, abnormal cytology, and positive ECC following surgery [11, 22, 23]. Intraoperative ECC is generally recognized as indicative of the status of the endocervical margin. In particular, in their study, Giannini et al. found that for patients with both positive surgical margins and HPV persistence, only positive endocervical margins, rather than ectocervical margins, were associated with worse outcomes [24]. However, only a few studies have been conducted on the relationships between preoperative abnormal ECC, postoperative lesions, and HPV status.

In a paired sample study in patients with recurrence/residual disease following LEEP, it was found during the postoperative review that abnormal preoperative ECC, positive resection margin, postoperative HR-HPV infection, and abnormal cytology were independent risk factors for positive ECC [9]. The status of the resection margin following LEEP may, however, directly affect the postoperative HR-HPV and cytology outcomes. The skipped distribution of the original lesions is the most likely cause of residual disease/recurrence with negative resection margins. In this study, we only included patients with negative margins during LEEP, which reduced the prognostic bias of margin factors. In our multivariable logistic regression analysis, we included variables such as menstrual status, ECC, and post-LEEP HPV that we selected using univariate analysis and found that both preoperative ECC and post-LEEP HPV at the 12-month follow-up were independent risk factors for residual disease/recurrence within 24 months.

We also found that, at 24 months following LEEP, the residual disease/recurrence rate was 9.3% higher for patients with HSIL and abnormal ECC than for those with normal ECC, while the risk of residual/recurrent disease was 4.06× greater. It is worth noting that the 2-year residual disease/recurrence rate of patients in the abnormal ECC group was 15.9%, which is higher than that previously reported (2.8–11.3%), while there was no significant difference in the HR-HPV negative conversion rate at the three follow-up timepoints. This indicates that patients with abnormal preoperative ECC, even if the LEEP margin is negative, are at a higher risk of residual disease/recurrence, and this risk may not be related to postoperative HR-HPV infection. One probable explanation is that there can be skipped lesions deep in the cervical canal, and this area can be easily overlooked during HR-HPV sampling. It is not known if increasing the cone height can reduce the risk of residual disease/recurrence in women with HSIL and abnormal ECC without fertility requirements, and this needs to be explored in future studies. Second, in this study, the negative conversion rate of HR-HPV at 6, 12, and 24 months post-surgery was consistent with the rates previously reported (67–86.7%, 78.9–96.5%, and 86–97.9%, respectively), but the rate of negative conversion of HR-HPV did not increase progressively after 12 months post-operation [25–28].

Some authors have noted that persistent HPV infection following LEEP may be related to the preoperative HPV viral load; however, in this paper, we focused on the HR-HPV type rather than viral load [26]. As a result, the preoperative viral load of patients with persistent HPV after 12–24 months might explain why the negative conversion rate did not improve with time.

This study has other limitations and deficiencies. Firstly, this study is a retrospective study, which has the inherent shortcomings of retrospective study, and further prospective multi-center study is needed. Thirdly, patients in this study were followed for only 24 months. We believe that adding additional follow-up time could yield more meaningful results.

## Conclusion

In conclusion, we found that women with HSIL and abnormal preoperative ECC were older, menopausal, had high-grade cytology, were infected with HPV 16/18, and had a type 3 TZ. Age and HPV 16/18 infection were independent risk factors for abnormal ECC. We suggest that preoperative ECC examination should focus on patients with the aforementioned characteristics since they may benefit more from it. Additionally, we found that abnormal preoperative ECC was one of the risk factors for post-LEEP residual/recurrent HSIL, with a 4.06 times higher risk of experiencing residual/recurrent disease. As a result, clinical attention to the detection of abnormal ECC can contribute to more focused management during follow-up.

## Data Availability

The raw data were obtained from the Department of Obstetrics and Gynecology, Renji Hospital, School of Medicine, Shanghai Jiaotong University. The data that support the findings of this study are available from the first author upon reasonable request.

## Abbreviations

HSIL: High-grade squamous intraepithelial lesion
ECC: Endocervical curettage
HPV: Human papillomavirus
LEEP: Loop electrosurgical excision procedure
TZ: Transformation zone
HR-HPV: High-risk HPV
SIL: Squamous intraepithelial lesions
TCT: Thinprep cytology test
